# Reducing the Amount of Catalyst in TEMPO-Oxidized Cellulose Nanofibers: Effect on Properties and Cost

**DOI:** 10.3390/polym9110557

**Published:** 2017-10-26

**Authors:** Albert Serra, Israel González, Helena Oliver-Ortega, Quim Tarrès, Marc Delgado-Aguilar, Pere Mutjé

**Affiliations:** Group LEPAMAP, Department of Chemical Engineering, University of Girona, c/M. Aurèlia Campmany, n° 61, Girona 17071, Spain; bertu.serrin@gmail.com (A.S.); helena.oliver@udg.edu (H.O.-O.); joaquimagusti.tarres@udg.edu (Q.T.); m.delgado@udg.edu (M.D.-A.); pere.mutje@udg.edu (P.M.)

**Keywords:** cellulose nanofibers, biopolymer, TEMPO oxidation, chemical properties, physical properties, nanopaper, mechanical properties

## Abstract

Cellulose nanofibers (CNF) are interesting biopolymers that find numerous applications in different scientific and technological fields. However, manufacturing costs are still one of the main drawbacks for the industrial production of highly fibrillated, transparent CNF suspensions. In the present study, cellulose nanofibers were produced from bleached eucalyptus pulp via TEMPO-mediated oxidation with varying amounts of NaClO and passed through a high-pressure homogenizer. The CNFs were chemically and physically characterized; cellulose nanopapers were also produced to study tensile properties. Production costs were also calculated. Results indicated that CNF properties are strongly dependent on the carboxyl content. Manufacturing costs showed that chemicals, in particular TEMPO catalyst, represent a large part of the final cost of CNFs. In order to solve this problem, a set of samples were prepared where the amount of TEMPO was gradually reduced. Characterization of samples prepared in this way showed that not only were the costs reduced, but also that the final properties of the CNFs were not significantly affected when the amount of TEMPO was reduced to half.

## 1. Introduction

The interest in cellulose nanofibers (CNFs) for scientific and technological applications has grown progressively during the last few years. Properties such as biodegradability, low density, good tensile strength and rigidity, biocompatibility and high availability make CNFs a target for researchers seeking versatile, novel biomaterials. Moreover, the fact that cellulose can be obtained from a variety of sustainable raw materials and the possibility of performing chemical modification on fibers, make it possible to modify the properties of CNF depending on the requirements and applications. Many industrial uses have been proposed for CNFs, among them, the papermaking [1,2,3,4,5,6], food [7,8] and packaging [9,10] industries have shown great advances in CNF applications. Also, the use of CNF in green electronics is being investigated [11,12,13].

The first attempts to produce CNF came from experiments where wood cellulose fiber suspensions were forced to pass through high pressure homogenizers. However, in order to effectively obtain nano-sized cellulose fibers it became necessary to pass the fibrous suspensions through the homogenizer many times, which resulted in large amounts of energy being applied. This heavily elevates production costs, making cellulose nanofibers impractical to be manufactured on an industrial scale [14]. Clogging of the equipment was also identified as a major problem when producing cellulose nanofibers. These disadvantages prompted researchers to look for alternatives to reduce production costs. Thus, a diversity of pretreatments was developed in order to ease and reduce the number of passes through homogenizers and microfluidizer equipment that were necessary to obtain CNF. Grinders, mills and high-speed mixers and even common blenders have also been used to mechanically delaminate cellulose fibers and release the nanofibers. Current pretreatments can be classified into three categories: chemical, enzymatic, and mechanical. Some typical chemical pretreatments include acid or alkaline hydrolysis [15,16,17], carboxyl methylation [18] and TEMPO-mediated oxidation [19,20,21], among others. Enzymatic hydrolysis comprises the use of cellulases, particularly endoglucanases, which depolymerize amorphous regions in the cellulose chain [22,23,24]. Mechanical pretreatments apply shearing forces to the fibers prior to the final delamination stage [25]. Depending on the type of pretreatment, the CNF’s final properties and costs can vary significantly.

Of all the pretreatments mentioned above, TEMPO-mediated oxidation has been a very popular method, at least at laboratory levels, during recent years. Basically, the reaction oxidizes the C6 of the d-glucose unit by substituting the –OH group of that carbon for a COOH group. The reaction uses either NaClO or NaClO_2_ as oxidizers in the presence of NaBr at alkaline conditions; the catalyst reagent TEMPO (2,2,6,6-tetramethylpiperidine-1-oxyl radical) and its analogues are water soluble, commercially available and stable nitroxyl radicals, most of which have a negative result for the Ames test [26], a standard test developed to determine the mutagenic potential of chemical compounds. The reaction can be carried out at room temperature and it takes between 1 and 2 h to complete depending on the final carboxyl content desired. TEMPO-mediated oxidations are usually applied to commercially-available bleached wood pulps [27,28,29], though other sources can also be used as raw material for CNF production [30,31,32].

Previous work by our research group has demonstrated that, although TEMPO-oxidized CNF presents high yields of fibrillation and transparency, the manufacturing prices can be higher than other types of CNF with different pretreatment methodologies [33]. This is contradictory since TEMPO-mediated oxidation, though it certainly helps to reduce the number of passes through a high-pressure homogenizer/microfluidizer, is a relatively expensive reactant whose price can easily surpass energy consumption costs. Therefore, TEMPO-mediated oxidation is not attractive to industrial manufacturing given the other alternatives.

In the present work, we study the production and characterization of cellulose nanofibers via TEMPO-mediated oxidation with different carboxyl content. Manufacturing costs at the laboratory level are also analyzed. Then, production experiments are repeated on different CNF batches where the quantity of TEMPO catalyst was gradually reduced. Production costs are recalculated and cationic demand studied as a way to evaluate the influence of lower TEMPO quantities on CNF properties. To the best of the authors’ knowledge, this is the first time that the influence of reducing the amount of TEMPO catalyst is studied as an alternative for reducing the costs of CNF prepared via TEMPO-mediated oxidation. Though the results presented in this work should not be considered as representative of industrial conditions, they certainly can be used as a starting point for further investigation on cost-properties optimization.

## 2. Materials and Methods 

**Materials.** CNF was prepared from commercial dried bleached *Eucalyptus globulus* pulp kindly provided by La Montañesa (Torraspapel Group, Zaragoza, Spain). TEMPO, sodium bromide (NaBr) and NaClO for the TEMPO mediated oxidation were obtained from Sigma-Aldrich, Barcelona, Spain and used as received. All suspensions were made in distilled water.

**Preparation of cellulose nanofibers.** In a typical experiment, 15 g of fibers were dispersed in distilled water containing previously dissolved TEMPO (0.016 g per g of fibers) and NaBr (0.1 g per g of fibers). The mixture was stirred for 15 min in order to assure good dispersion of all the substances. After this, a 15% NaClO solution was added drop-wise to the slurry. The volume of NaClO was calculated to add 2, 3, 4, 5, 6, 8, 10 and 15 mmol of NaClO per g of cellulose in different batches. The pH was kept at 10 by the addition of a 0.5 M NaOH solution. The oxidation was finished when the pH stayed constant at 10. The oxidized fibers were then filtered and washed with distilled water. A 1–2 wt % water suspension of oxidized fibers was passed through a high-pressure homogenizer (NS1001L PANDA 2000-GEA, GEA NIRO, Parma, Italy) operating at 600 bar pressure. The process was repeated six times. The resulting gel-like suspension was then preserved at 4 °C. Depending on the mmol of NaClO added, samples were named as CNF-2, 3, 4, 5, 6, 8, 10 and 15.

**Characterization of cellulose nanofibers.** The degree of polymerization was determined from intrinsic viscosity measurements, according to UNE 57-039-92. The viscosimetric average molecular weight was calculated from the equation: η = *K*·*M^a^*, where η is the intrinsic viscosity (dL/g), *K* and *a* are constants (2.28 and 0.76 respectively), which depend on the polymer-solvent system; and *M* is the molecular weight. Details of the equation and the constants used in this work have been taken from Henriksson et al. [34]. 

Yield of fibrillation was determined by centrifuging a 0.2% solid content CNF suspension at 4500 rpm for 20 min in order to separate the nanometric fraction (retained in the supernatant) from non-nanometric solids which remain at the bottom of the sample container. The solids were removed from the sample container, weighed and oven-dried at 105 °C until a constant weight. The yield of nanofibrillation was then calculated using the equation:

Yield (%) = (1 − weight of dried sediment/weight of diluted sample × %Sc) × 100(1)

where %Sc represents the solid content of the diluted CNF sample. The water retention value (WRV), was calculated by separating a given volume of CNF gel into 2 identical portions, which were then centrifuged in a Sigma Laborzentrifugen model 6K15 (SIGMA, Osterode am Harz, Germany) at 2400 rpm for 30 min to eliminate non-bonded water. CNFs were retained with a nitrocellulose membrane with a pore size of 0.65 μm diameter set at the bottom of the centrifuge bottles. After centrifugation, only the CNF in contact with the membrane was recovered, weighed and then dried at 105 °C for 24 h in previously weighed bottles. This methodology is based on TAPPI UM 256. The average WRV was finally calculated according to the equation:

WRV (%) = (*W*_w_ − *W*_d_/*W*_d_) × 100 (2)

where *W*_w_ is the wet weight (g), *W*_d_ the dry weight (g). The carboxyl content of oxidized cellulose fibers was determined by conductometric titration. The dried sample (50–100 mg) was suspended in 15 mL of 0.01 M HCl; this exchanges Na cations bound to the COOH group by H ions. After 10 min of mechanical mixing, the suspension was titrated with 0.01 M NaOH, adding 0.1 mL of NaOH to the suspension, and recording the conductivity in mS/cm; this process was repeated until observation of a reduction, stabilization and increase in conductivity. With these results, a conductometric titration curve was constructed, showing the presence of a strong acid that corresponds to the excess of HCl and a weak acid that corresponds to the carboxyl content. The carboxyl content (*CC*) is given by the following equation:

*CC* = 162(*V*_2_ − *V*_1_) *c* [(*w* − 36(*V*_2_ − *V*_1_))] (3)

where *V*_1_ and *V*_2_ are the equivalent volumes of the added NaOH solution (L), *c* is the NaOH concentration (mol/L), and *w* the weight of the oven-dried sample (g). The results indicate the average mmol of –COOH groups per gram of CNF. Details of this technique are given by Da Silva et al. [19].

The cationic demand was determined with a Mütek PCD 04 particle charge detector (BTG Mütek GmbH, Herrsching, Germany). First, 0.04 g of CNF (dried weight) was diluted in 1 L distilled water and dispersed with a pulp disintegrator for 10 min at 3000 rpm. Next, 10 mL was taken and mixed with 25 mL of cationic polymer polydiallyldimethylammonium chloride (polyDADMAC) for 5 min with magnetic stirring. After this, the mixture was centrifuged in a Sigma Laborzentrifugen model 6K15 (SIGMA, Osterode am Harz, Germany) for 90 min at 4000 rpm. Subsequently, 10 mL of the supernatant was taken to the Mütek equipment where anionic polymer (Pes-Na) was added drop by drop until the equipment reached 0 mV. The volume of Pes-Na consumed was written down and used to calculate the cationic demand (eq/L, meq/L, μeq/L) via the following equation:

Cationic demand = (*C*_poly-D_ · *V*_poly-D_) − (*C*_pes-Na_ · *V*_pes-Na_)/W_sample_ (4)

where *C*_poly-D_ and *V*_poly-D_ are the concentration and volume of poly-DADMAC, respectively; likewise, *C*_pes-Na_ and *V*_pes-Na_ are the concentration and volume of pes-Na polyelectrolyte; *W*_sample_ is the sample weight (g). Details of this methodology have been also published elsewhere [25]. Specific surface and diameter were estimated from the carboxyl content and cationic demand. For this, it was considered that the interaction between the CNF surface, which has hydroxyl and carboxyl groups, and the added cationic polymer happened through two unlike mechanisms: on the one hand, part of the polymer was held through ionic interaction between carboxyl groups from CNF and the polymer thereof. On the other hand, the rest of the consumed poly-DADMAC during cationic demand analysis was assumed to be retained by surface adsorption. Furthermore, the calculation of the specific surface area was accomplished taking into account two suppositions: (1) the surface adsorption of poly-DADMAC takes place as a monolayer, and (2) the poly-DADMAC has a cylindrical geometry. The surface of poly-DADMAC was first estimated by calculating the monomer’s surface and its degree of polymerization, considering the bond distances and assuming a cylindrical shape. The calculated dimensions were 5.427 Å for the diameter and 4.849 Å in length, obtaining a surface value of 535.87 nm^2^, equivalent to 4.874 × 10^17^ nm/μeq, according to Espinosa et al. [31]. Using the values obtained for cationic demand and the carboxyl content it was possible to calculate the CNF’s theoretical surface area by using the following equation:

σ_CNF_ = (*CD* − *CC*) · *S*_poly-D_ (5)

where *CD* is cationic demand, *CC* is carboxyl content (both expressed in µeq-g/g) and *S*_poly-D_ is the specific surface of poly-DADMAC, in nm^2^/µeq-g. Finally, assuming that nanofibers are cylinders and that cellulose density is 1.5 g/cm^3^, diameter was calculated through the next equation:

*m*^2^⁄*g* = 4/*ρ* · *d* (6)

where *ρ* is cellulose density and d is the diameter of a single nanofiber. By calculating for *d*, it is then possible to obtain the theoretical diameter of a single cellulose nanofiber. More details about this technique have already been published elsewhere [31]. The production costs of CNF at laboratory level were calculated by adding the energy consumption of the equipment used for CNF manufacturing and the price of chemicals acquired in the Spanish market. Energy consumption was determined using energy measuring equipment Circutor CVM-C10 (CIRCUTOR, Viladecavalls (Barcelona), Spain) and a Socomec Diris A20 (GRUPO SOCOMEC, Badalona (Barcelona), Spain). The cost of energy was estimated at 0.08 €/kWh.

**Nanopaper production.** Cellulose nanopapers were produced via a modified laboratory paper sheet former. In a typical experiment, 152 g of 1 wt % CNF gel was diluted with water until reaching 0.2 wt % of consistency (760 g wet weight). The suspension was then dispersed by stirring with an Ultraturrax T25 (IKA, Staufen im Breisgau, Germany) at 20,000 rpm for 2 min. Next, the suspension was poured into a sheet former with a 0.22 μm nitrocellulose membrane at the bottom of the stock container. The suspension was vacuum filtered until a gel-like cake was formed on top of the membrane. The cake was then removed and vacuum dried for 15 min. The resulting nanopapers were finally stored at 23 °C and 50% of relative humidity for 24 h before testing.

**Characterization of nanopapers.** Nanopaper samples were mechanically tested in an Instron universal testing machine (INSTRON, Barcelona, Spain) provided with 2.5 kN load cell. Nanopaper samples were carefully cut down to strips of 180 × 15 mm. Distance between gaps was set at 150 mm, preload was set at 0.1 N, with a cross-head velocity of 15 mm/min. Transmittance measurements were performed on a UV-Vis Shimadzu spectrophotometer UV-160A (Shimadzu Europa GmbH, Duisburg, Germany) set in the range between 400 and 800 nm. The samples were introduced without cuvettes and using air as a reference. Porosity was determined from the density of the nanopaper by applying the next equation:

Porosity (%) = 100 · (1 − *ρ*_sample_/*ρ*_cellulose_) (7)

where *ρ*_sample_ is the density of the biocomposites and *ρ*_cellulose_ is the density of pure cellulose, assumed to be 1.5 gr/cm^3^. Nanopaper density was calculated from the base weight and the sample thickness. FE-SEM microphotographs were taken with a Hitachi S-3000 (Hitachi Europe S.A., Barcelona, Spain) working at 12 kV; samples were previously covered with carbon via sputtering.

## 3. Results

### 3.1. Chemical Properties

Table 1 shows the development of the chemical properties of the CNF in relation to the carboxylic content. The number used for naming each sample (except the original pulp) indicates the amount of NaClO in mmol used during TEMPO-oxidation. The first result column presents the amount of carboxyl groups detected in each sample. In this work, the amount of COOH groups per gram of cellulose was determined via two methodologies: the conductivity method and methylene blue. Both methodologies yield similar results, though it is known that the methylene blue technique tends to underestimate the real amount of carboxylic groups in comparison to conductivity methods, especially for low DP fractions [19].

Such differences are more noticeable in samples CNF-3 and CNF-4, while the variances are less significant in the rest of the samples. Carboxyl content in CNF increases when more NaClO is added during the TEMPO-mediated oxidation, which also brings about a boost in the cationic demand of the CNF suspension. The increase in carboxyl content is important when we compare the figures of the original pulp and the rest of the samples. One of the main advantages recognized in TEMPO-mediated oxidation of cellulose fibers was that the introduction of carboxyl groups facilitates the release of the cellulose microfibrils, the fundamental structure that forms the hierarchical organization of cellulose fibers. It is important to note that the term “microfibril” is used for single nanometric fibers with diameters of around 3–5 nm, whereas “nanofibers” can refer either to single microfibrils or bundles of microfibrils whose diameters are still in the nanometric scale. Carboxyl groups on the cellulose surface increase the overall surface’s negative charge, which generates repulsion between fibers. The hydrophilicity of the fibers is also enhanced. When TEMPO-oxidized fibers are exposed to shearing, high impact forces like those present in a high-pressure homogenizer or a microfluidizer, the nanofibers are released, modifying the overall suspension rheology. This type of chemical pretreatment greatly reduces the number of passes through a homogenizer that would be required for non-treated fibers. Less passes mean lower energy costs.

**Figure 1:**
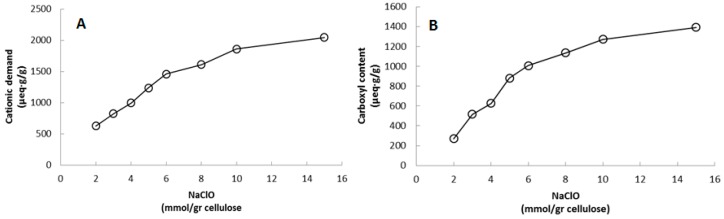
Increase of cationic demand (**A**) and carboxyl content (**B**) in relation to the amount of NaClO added during the TEMPO-mediated oxidation.

Cationic demand is a parameter used in papermaking characterization to indirectly determine the specific surface area of a cellulose fiber suspension. Highly individualized fibers (high degree of fibrillation) translate into a large specific surface area which exposes most of the hydroxyl groups on the surface of the cellulose fiber. This increases the amount of cationic polymer necessary to neutralize the suspension’s surface. Table 1 shows that cationic demand increases in relation to the amount of NaClO added during the reaction; in fact, this increase shows a linear correlation from samples CNF-2 to CNF-6 mmol of NaClO added (Figure 1). From CNF-8 to CNF-15 the trend line becomes less linear. This is because, as the TEMPO-mediated oxidation advances, less hydroxyl groups remain available to be converted into carboxyl ones. Therefore, the increase in cationic demand at higher NaClO content will be more restrained than at lower content levels (Figure 1). Besides, it is well known that cellulose is partially depolymerized during the TEMPO reaction, forming water-soluble saccharides that are washed away during the rinsing stage of the typical CNF preparation methodology. This reduces the original amount of oxidized cellulose and therefore reduces increases in cationic demand as carboxyl content intensifies.

**Figure 2:**
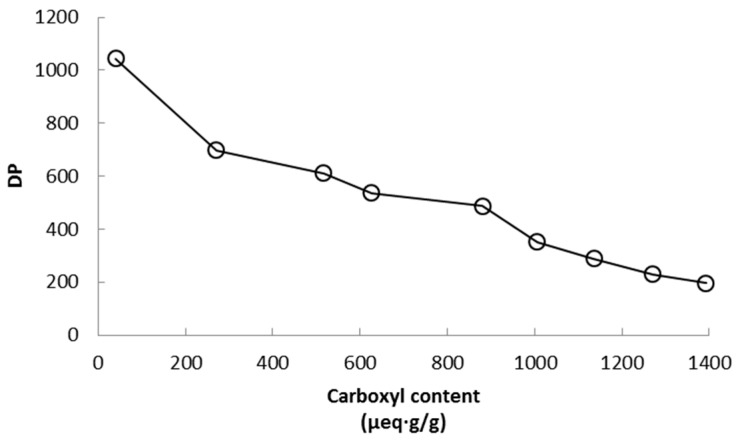
Decrease of the degree of polymerization in relation to the carboxyl content of cellulose nanofibers.

The degree of polymerization (DP) of cellulose fibers suffers an important reduction that depends on the amount of NaClO added (Figure 2). Depolymerization of TEMPO-oxidized cellulose fibers is a typical feature when the reaction is carried out at alkaline conditions, as in the present work. Though TEMPO-mediated oxidations at neutral pH produce low depolymerization, such oxidations require more time to react and consume both sodium chlorite and hypochlorite. The lowest degree of polymerization is seen in sample CNF-15 which was expected. Two possible mechanisms have been proposed to explain the depolymerization of cellulose during the TEMPO-mediated oxidation at alkaline conditions: first, β-elimination due to the formation of aldehyde groups on the C6 as intermediate structures at alkaline pH; and second, cleavage of the (1-4)-β-glycoside bonds of the cellulose chain by active radical species that appear as a result of lateral reactions that occur during the TEMPO-mediated oxidation [26]. It is important to note that these results correspond to CNF before the homogenization step, therefore it is expected that further depolymerization occurred during the mechanical treatment of the oxidized fibers. Moreover, another slight depolymerization may also occur during the dissolution of CNF in cupryethylenediamine used for the viscosity assay due to the presence of residual aldehyde groups on the C6. Though TEMPO-oxidations at neutral pH produce moderate depolymerization, the rate of conversion from hydroxyl to carboxyl groups during the reaction has been reported to be lower in comparison to alkaline conditions [26]. In fact, Shinoda et al. [35] found a good relationship between DP and nanofiber length by plotting viscosimetric DP values against CNF lengths observed by electronic microscopy. It can be expected that a good part of the depolymerization process occurs in the amorphous zones of the cellulose chain.

### 3.2. Physical Properties

Table 2 shows the results for the physical characterization of CNFs. The first result column presents the yield of fibrillation of every CNF suspension. TEMPO-oxidized CNFs already present high values at low carboxyl content; the yield of fibrillation represents the percentage of solid material within the nanometric scale. Values of almost 100% are observed in samples CNF-8 through CNF-15. The yield of fibrillation also demonstrates that the amount of carboxyl groups in cellulose determines the extent of microfibrils release from the cellulose fiber’s main structure during the mechanical treatment stage. This parameter relies on the assumption that the amount of nanofibers in the supernatant after centrifugation increases as the fibrillation process becomes more efficient [36]. Since highly charged fibers are expected to withstand better centrifugation, dilutions of CNF with high carboxyl content are expected to present high degrees of fibrillation.

Transmittance at 600 nm also evolves positively from sample CNF-2 through CNF-15. Transmittance is highly dependent on the fiber diameter within the water suspension. CNF with diameters below the wavelength of visible light, lose capacity to scatter light, therefore CNF suspensions with high yield of fibrillation at the same concentration will present higher transmittance than their counterparts with lower yields. CNF-15 presented the highest transmittance among all of the samples analyzed. The high density of carboxyl groups in the samples, produces repulsion between the fibers suspended in water which as a result gives homogeneous, almost transparent CNF dispersions with low light-scattering properties. Lower transmittance results in sample suspensions with low carboxyl content are due to the presence of nanofiber aggregates.

Water retention value (*WRV*) was also studied. This parameter is also typical in the papermaking industry and determines the ability of fibers to take up water and swell. This parameter also increases with the specific surface area in fibers. Sample CNF-15 showed the highest *WVR*, which is in accordance to the other properties that also increase with carboxyl content. Compared to the initial WRV of 3.9, sample CNF-15 presents an increase of 192%.

Specific surface area is also strongly dependent on the carboxyl content. In this paper, the surface area of CNF was calculated from the cationic demand, carboxylic content and the surface area of cationic polymer poly-DADMAC. The results indicate that samples CNF-2 through CNF-5 present, in general, similar specific surfaces, except for sample CNF-3 which was somehow lower than the rest of that particular set of samples. For samples CNF-6 through CNF-15, a steady increase is observed. In the same way, the theoretical diameter of CNF shows similar results in samples CNF-2 to CNF-5, averaging diameters of 15 nm. This clearly corresponds to bundles of microfibrils that have not been completely individualized during the homogenization process. The rest of the samples exhibited lower diameters, with sample CNF-15 presenting the smallest diameter. Though specific surface area and diameter are theoretically calculated, they are a good indicator of the influence that carboxyl content has on the CNF morphology and the possibility of adjusting such properties by controlling the addition of oxidant.

### 3.3. Production Costs

Table 3 shows the production costs for each type of CNF prepared in the present study. The results effectively show that adding more NaClO during TEMPO-mediated oxidation helps to reduce the overall energy consumption. The second column of results from left to right represents the energy consumed for stirring the fiber slurry during the TEMPO reaction. The third column indicates the energy consumption during the homogenization. From sample CNF-2 through sample CNF-15 there is a total cost reduction of € 5.65. Nevertheless, the results also indicate, that in order to reduce the energy costs it becomes necessary to increase the quantity of NaClO. The amount of chemicals required depends of course on the yield of fibrillation needed. Though chemical pretreatment is not the only method commonly used for CNF manufacturing, it produces highly individualized, transparent CNF gels. In order to find a way to further reduce the costs of chemicals, a set of samples was prepared where the amount of TEMPO added was progressively reduced. This was because TEMPO is the most expensive chemical from all the reactants used for the oxidation. In order to observe how this affected CNF properties, cationic demand of the samples was also studied as an indicator of CNF quality. Table 4 shows the evolution in price per kg of CNF (dried weight) as TEMPO is progressively reduced. The amount of NaClO was calculated to obtained sample CNF-5. The table also includes the cationic demand for every sample as an easy way of determining the fibrillation degree of the samples.

The column of chemicals describes the price of all the chemicals used during the reaction, whereas the column, total cost refers to the final price (including both chemicals and energy consumption) per kg of CNF. The starting amount of TEMPO used for a normal reaction (16 g/kg of CNF) corresponds to 1 mol of TEMPO for 1 mol of cellulose. The progressive reduction in the amount of TEMPO catalyst reduces the overall cost of CNF-5 from 8.74 to 6.89 €/kg, which represents a reduction in final price of 21%. It is interesting to note that cationic demand remains approximately the same when the amount of TEMPO is reduced from 16 to 2 g/kg of CNF. This is an important feature since most studies dealing with TEMPO-oxidized cellulose nanofibers do not consider lesser quantities of chemicals for the optimization of CNF prices.

The only drawback to this alternative is that oxidation times become longer when the amount of catalyst is considerably reduced. When the initial stoichiometry relationship of 1 mol of TEMPO per mol of cellulose is kept, the reaction (for 5 mmol of NaClO) requires around 1 h to be completed. When the quantity of TEMPO was halved, the reaction time remained approximately the same, but when the amount of TEMPO was 4 and 2 g/kg of CNF, the reaction required up to two hours until all the NaClO was consumed. Finally, the reaction required 5 h to be completed when the presence of TEMPO was reduced to only 0.5 gr/kg of cellulose. Longer times are required when the amount of NaClO to be oxidized increases.

### 3.4. Mechanical and Physical Properties of Nanopapers

Cellulose nanopapers were prepared via casting technique in order to understand how carboxyl content affects the mechanical and physical properties of dried CNF. Table 5 presents the results for samples CNF-2, CNF-6, CNF-10 and CNF-15. Tensile strength was significantly enhanced with the carboxylic content. In fact, there is a linear relationship between the carboxylic content and the tensile strength. Regarding Young’s modulus, increases were much more discreet, though the best result was again found in sample CNF-15. It is interesting to note, that strain at break also increased with more carboxyl content. The improvement of these properties can be related to the different degrees of fibrillation from one sample to another. It is well known that strength in ordinary paper depends mainly on the number of hydrogen bonds that form among cellulose fibers when water is removed from a fibrous suspension. Following this reasoning, it can be expected that cellulose nanofibers with a very high aspect ratio and specific surface area will be able to form large amounts of hydrogen bonds between them. The second parameter that controls paper strength is the intrinsic strength of the fibers. It is expected, however, that individual cellulose nanofibers do not perform to their full potential due to possible damage that occurs during chemical pretreatment and mechanical disintegration, a similar trend observed in cellulose fibers that are submitted to intense chemical treatments [37]. Furthermore, the homogenous morphology of oxidized CNF helps to form nanopapers with uniform, compact structures. This combination of properties produces a better distribution of stress throughout the nanopaper during the tensile test.

**Figure 3:**
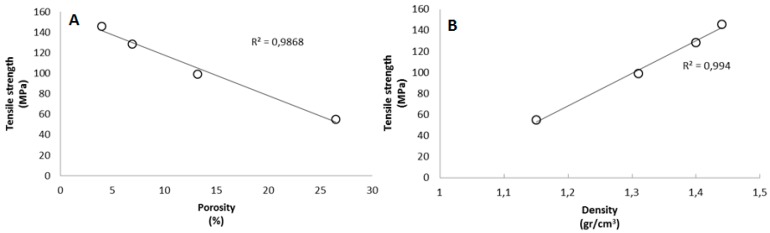
Relationship between tensile strength and (**A**) porosity; (**B**) density.

The improvement in the nanopaper structure can also be assessed by observing the results obtained for porosity and density. In the case of porosity, calculated as the percentage of free space among fibers, it decreases considerably from sample CNF-2 to CNF-15, meaning that nanopapers become more compact at higher carboxyl content. The enhancement in compactness means that density also increases according to carboxyl content. In fact, the value of 1.44 gr/cm^3^ of sample CNF-15 is close to the density of pure cellulose (1.5 gr/cm^3^). Several works have also remarked on the significant dependence of the mechanical properties on the degree of polymerization. Cellulose nanopapers based on CNF with low DP are in general weaker than those with higher DP [34]. Though the results in the present work contradict this fact, it is also true that the expansion in the number of hydrogen bonds and increased compactness in nanopapers can compensate for the expected loss in mechanical properties due to high carboxylic content. Previous works have already demonstrated that good mechanical properties can still be obtained at relatively low DP [38]. In fact, it is possible to observe a very good linear correlation between the tensile strength and density (Figure 3).

**Figure 4:**
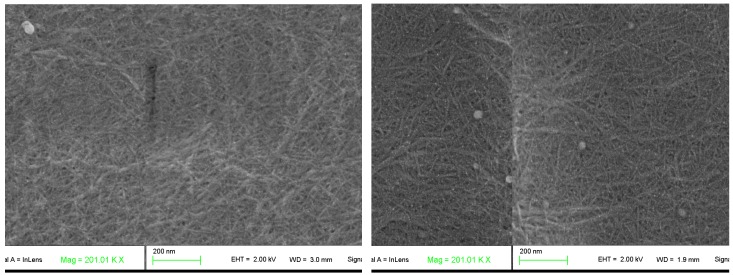
FE-SEM microphotography of cellulose nanopapers prepared from CNF at 6 (**left picture**) and 10 mmols of NaClO (**right picture**).

Field-emission SEM microphotography performed on nanopapers prepared from samples CNF-6 and CNF-10 (Figure 4) show the overall assembly of nanopapers. Both images show similar structures where nanofibers are randomly entangled where no nanofiber ends can be discerned. Empty spaces are also observed in some areas among the nanofibers, whereas other zones are well compacted and show no visible pores. The nanofiber diameter is around 10 nm in both samples, though it is also possible to see bundles of nanofibers with larger diameters. It is important to note that the diameters observed in these two samples are very similar to those predicted theoretically.

## 4. Discussion

Chemical and physical properties of cellulose nanofibers produced via TEMPO-mediated oxidation depend on the carboxyl content and reaction conditions. This can be stated since all the samples were passed through the high-pressure homogenizer six times, meaning that the same amount of energy was applied in all cases. The introduction of charged groups to the surface of cellulose as a fast, effective way to facilitate CNF production has already been demonstrated by many authors [19,20,28,39,40]. One of the main advantages offered by CNF manufactured through this type of methodology, is its high transparency. Since TEMPO-oxidized CNFs achieve diameters between 5 and 100 nm, their aqueous suspension and films present elevated transmittance; this is a desirable property for applications where transparency is necessary [41]. Cellulose nanopapers with highly fibrillated CNF also show very low porosity, a property that is of interest in gas-barrier applications and functionalized membranes [42,43].

Though the introduction of negatively charged groups on the cellulose surface produces high defibrillation, the overall cost of the reactants does not compensate for the energy savings. This is an important disadvantage for CNF-based materials if we compare the costs of well-established, transparent materials such as polyethylene terephthalate PET, which shows some properties similar to those of cellulose nanopaper from TEMPO-CNF, but at a much more competitive price [12]. The reduction in the amount of TEMPO catalyst stands as an alternative. By halving the quantity of catalyst we observed a cost reduction of 11%; further reduction in the amount of the catalyst up to 2 g/kg of fibers brought about savings of up to 19%. Cationic demand was not significantly affected by the reduction of TEMPO. In fact, that parameter remained almost constant and a meaningful decrease was not observed until samples were treated with only 0.5 g of TEMPO per kg of cellulose. However, by reducing the amount of TEMPO, reactions took longer to complete, even though reaction times of about 2 h could be considered as normal.

It is interesting to note that in the present work, the mechanical properties of nanopapers did not diminish at low degrees of polymerization, rather the opposite. This result strongly contrasts with what is usually found in the literature where good relationships between DP and tensile strength can be drawn [34]. A possible explanation could be that the high specific surface area available in TEMPO-oxidized CNF greatly enhances the number of hydrogen bonds (the main mechanism that governs paper strength) between adjacent fibers, which eventually compensates the loss in tensile strength due to the decrease in DP. In fact, the accepted tensile strength of cellulose nanopapers of 200–250 MPa is reported for CNF with a DP around 1000 [34,44], and such values could not be achieved in the present work. The literature reports that the final DP of TEMPO-oxidized fibers depends more on reaction conditions than carboxyl content. For example, fibers with similar final carboxyl content oxidized at alkaline conditions present lower DP than those treated at neutral pH [45]. This has been attributed to side reactions that depolymerize the cellulose chain under alkaline conditions [21,26,45]. However, neutral conditions require longer reaction times (more than 20 h depending on required carboxyl content) and moderate temperature (60 °C) before the reaction is completed.

Though prices of cellulose nanofibers produced via TEMPO oxidation are still well above those of other materials such as most plastics, it is important to remember that cellulose has other advantageous properties such as renewability, a low thermal expansion coefficient and high Young’s modulus, characteristics that cannot be found in many commodity plastics. More research is necessary in order to further reduce the production costs of TEMPO-CNF since this particular type of nanofiber presents interesting properties with numerous possible applications in a wide variety of technological areas.

## 5. Conclusions

In the present work, cellulose nanofibers with different carboxyl content were prepared via TEMPO-mediated oxidation and high-pressure homogenization. Characterization included chemical, physical and tensile properties. Manufacturing costs at laboratory level were also studied. The resulting CNF was then used to prepare cellulose nanopapers in order to assess the mechanical and physical properties of the dried materials. The results indicated that carboxyl content and reaction conditions govern the final properties of CNF and this allows for tuning various characteristics to obtain CNFs with specific features. Economic analysis revealed that, although chemical pretreatment can certainly reduce energy consumption, the cost of reactants can be so high that it negates any saving in energy. However, this can be compensated for by reducing the amount of TEMPO catalyst. Studies in cationic demand demonstrated that by reducing the quantity of TEMPO to half the original amount, it is possible to obtain cellulose nanofibers with the expected cationic demand. Further reduction in TEMPO quantities also delivers CNF with high cationic demand, though oxidation reactions can take double the time to complete. TEMPO amounts of less than 1 g/kg of cellulose required up to 5 h and the resulting CNF presented lower cationic demand values which correspond to lower carboxyl content. The overall economic analysis indicates that it is possible to compensate for the escalation of costs due to chemicals by reducing the amount of TEMPO, which stands as the most expensive reactant used for the oxidation of cellulose while maintaining CNF quality. However, such results should be regarded with caution since other parameters must be considered when talking about industrial scaling.

## Figures and Tables

**Table 1:** Chemical properties of cellulose nanofiber (CNF) suspensions at different carboxylic content.

Sample	Carboxylic Content (µeq·g/g)		Cationic Demand (µeq·g/g)	Degree of Polymerization
	Conductivity Method	Methylene Blue Method		
Euc pulp	40	38	70	1045
CNF-2	271	247	625	698
CNF-3	517	312	824	611
CNF-4	626	453	996	536
CNF-5	881	801	1235	488
CNF-6	1006	990	1459	352
CNF-8	1136	1138	1611	289
CNF-10	1271	1157	1858	229
CNF-15	1392	1375	2043	197

**Table 2:** Physical properties of cellulose nanofibers at different carboxyl content.

Sample	Yield of Fibrillation (%)	Transmittance at 600 nm (%)	Water Retention Value (g/g)	Specific Surface (m^2^/g)	Diameter (nm)
CNF-2	90.02 ± 0.3	59	3.9 ± 0.3	172.4	14.50
CNF-3	91.27 ± 0.5	66	4.8 ± 0.5	149.5	16.72
CNF-4	92.53 ± 0.3	68	6.8 ± 0.2	180.2	13.87
CNF-5	93.01 ± 0.2	70	8.2 ± 0.3	172.4	14.50
CNF-6	94.87 ± 0.4	76	8.4 ± 0.1	220.6	11.33
CNF-8	97.03 ± 0.3	78	9.3 ± 0.4	231.3	10.81
CNF-10	97.69 ± 0.1	80	10.8 ± 0.2	285.9	8.75
CNF-15	98.88 ± 0.4	83	11.4 ± 0.4	317.0	7.89

**Table 3:** Production costs of cellulose nanofibers at different carboxylic content (laboratory level). The price of energy was 0.08 €/kW·h, price of NaClO was 0.38 €/kg, prices correspond to the Spanish market.

Sample	Chemicals Cost (€/kg)	Energy Cost (€/kg)	Energy Consumption (€/kg)	Total Cost (€/kg)
CNF-2	2.78	0.46	11.7	14.94
CNF-3	3.02	0.47	9.36	12.85
CNF-4	3.27	0.49	6.24	10.00
CNF-5	3.52	0.54	4.68	8.74
CNF-6	3.77	0.61	3.90	8.28
CNF-8	4.26	0.79	3.12	8.17
CNF-10	4.76	1.04	2.34	8.14
CNF-15	6	1.73	1.56	9.29

**Table 4:** Production costs of CNF-5 after progressive reduction in TEMPO amount.

Sample	TEMPO (g/kg of CNF)	Chemicals (€/kg of CNF)	Total Cost (€/kg CNF)	Cost Reduction (%)	Cationic Demand (µeq·g/g)
**CNF-5**	16	3.52	8.74	-	1547
	8	2.56	7.78	10.98	1280
	4	2.08	7.30	16.48	1557
	2	1.85	7.07	19.11	1504
	1	1.73	6.95	20.48	1410
	0.5	1.67	6.89	21.17	891

**Table 5:** Mechanical properties of cellulose nanopapers prepared from CNF with 4 different carboxylic contents.

Sample	σ_t_^NP^ (MPa)	ε_t_^NP^ (%)	*E*_t_^NP^ (GPa)	Transmittance at 700 nm (%)	Porosity (%)	Density (g/cm^3^)
CNF-2	54.7 ± 1.8	1.5 ± 0.08	11.05 ± 0.5	27.6 ± 1.05	26.5 ± 2.3	1.15
CNF-6	99.1 ± 3.2	1.98 ± 0.12	11.97 ± 0.3	52.3 ± 1.1	13.2 ± 1.9	1.31
CNF-10	128.3 ± 2.4	2.38 ± 0.14	12.40 ± 1.0	71.5 ± 1.9	6.9 ± 1.8	1.40
CNF-15	145.9 ± 4.3	2.60 ± 0.07	13.0 ± 0.5	79.9 ± 1.5	4.0 ± 2.0	1.44
